# Supplementary material for: An interactive geographic information system to inform optimal locations for healthcare services
Source: PLOS Digit Health. 2023 May 8;2(5):e0000253. doi: 10.1371/journal.pdig.0000253 (PMC10166531; doi:10.1371/journal.pdig.0000253)
Supplement: Attachment — Submitted filename: response_letter_20230124.docx [file pdig.0000253.s001.docx]

24 January 2023

Dear Editors and Reviewers,

**RE: Response to Reviewer Comments for “An Interactive Geographic Information System to Determine Optimal Locations for Healthcare Services” (PDIG-D-22-00277)**

Thank you for having reviewed our manuscript. We welcome the feedback supplied and have addressed each of these comments in the table below and have listed the relevant sections in the manuscript where changes have been made. Please note, we have also changed the title based on the reviewer feedback.

**Journal Requirements**

| Comment | Response | Submission/Manuscript Changes |
| --- | --- | --- |
| Please amend your online Financial Disclosure statement. | The requested changes have been made. | Changed in online submission |
| We ask that a manuscript source file is provided at Revision. | A source file has been provided in .docx, tracked and clean versions included. | Relevant files uploaded to online submission |
| Please provide separate figure files in .tif or .eps format only and remove any figures embedded in your manuscript file. | All figures have been provided in .tiff after verification with the Picture Analysis Conversion Engine (PACE). A separate .docx file has been provided with figure legends. | Relevant files uploaded to online submission |
| Figures 2, 3 and 4: please (a) provide a direct link to the base layer of the map (i.e., the country or region border shape) and ensure this is also included in the figure legend; and (b) provide a link to the terms of use / license information for the base layer image or shapefile. | All base layers have been sourced from OpenStreetMap, which is distributed under the Open Data Commons Open Database License. All shape files are accessed from the Australian Bureau of Statistics’ Australia Statistical Geographic Standard provided under a Creative Commons Attribution 4.0 International licence. | The details about the base map and shape files have been added to the figure 2 legend, details for the shape files have been added to the figure 3 and 4 legends (no base map in these figures). This has also been detailed in the Methods section at line 118 – 124. |

**Reviewer 1**

| Comment | Response | Manuscript Changes |
| --- | --- | --- |
| Would the authors provide more insight into why this particular study window was selected? (2000 – 2022) | The patient dataset used for this study is managed in-house by the Adult Congenital Heart Disease Service at Royal Prince Alfred Hospital; the year 2000 was chosen since this was the date from which uniform data collection of this dataset began. This uniform approach has been maintained since 2000 until the present day. | No changes have been made to the manuscript, regarding this point |
| Did the researchers conduct any statistical tests comparing changes in driving time before and after the hypothetical new clinics are added? | This project seeks to demonstrate the feasibility of an application that can demonstrate driving time difference between current clinics and new clinics to determine accessibility of clinics. The aim of this study is not to undertake a statistical analysis of the outputs of the clinical planning tool, but to demonstrate the feasibility of its development and show how that data can be used to optimise service provision. | No changes have been made |
| A little more context on what a clinic is and where it fits into the overall local health system (relative to a hospital/ large medical center) would be helpful for those who are not familiar with the Australian health system | ACHD clinics in New South Wales are medical services that are provided by ACHD-trained cardiologists and allied health staff. In New South Wales, this is run by a model of urban services with rural outreach, whereby a comprehensive ACHD centre exists at Royal Prince Alfred Hospital in Sydney, and several rural outreach clinics are run at various hospitals around New South Wales. ACHD-cardiologists from RPA will travel to these rural hospitals to run clinics for the local patient populations | Extra detail has been added to the introduction at lines 104 – 108, to clarify this aspect. |
| In the “Area Demographics Around Selected Hospital”, the authors described the IRSD scores of the selected areas for new clinics, and also described their ethnic composition. Would the authors also be able to present this information for the entire NSW area in addition to the demographic info (i.e. age, sex, etc.) presented earlier in the results? | In the “Patient Demographics” section, 2016 Census details have been included to outline demographic details for the state of NSW. The IRSD is a summary value that is calculated for smaller areas (like SA2) but not for the whole state of NSW and therefore cannot be reported in this way. This whole section’s values have been changed to reflect only the population that is currently alive, which matches the default settings of the app and thus better reflects the population used in the examples in the results section. | This is now clarified in lines 195 - 197 |
| Also, would the authors be able to include a results table with the demographic and CHD information for the study population? | A table showing a demographic and diagnosis summary of the study population has now been included, as suggested, at the beginning of the results section | Line 198 |
| Can the authors speak to if there are differences in CHD prevalence between areas with greater access to healthcare? | There are no such differences in prevalence; in NSW, there is no documented relationship between CHD prevalence and access to healthcare | No manuscript changes |

**Reviewer 2**

| Comment | Response | Manuscript Changes |
| --- | --- | --- |
| if the authors reconceive their work in terms of health access analytics and emphasize the ways that a) this type of approach compares to the *many* other examples of healthcare access GIS, an | Thank you for pointing out concerns about how this work compares with the published literature, with examples using the same software tools of R, shiny and leaflet, and other examples such as those provided by ERSI. We have provided a more detailed literature review of the most relevant examples of healthcare access GIS, and emphasise the main novelty of our current approach; which is the novel concept of using such data for future service planning (see our response immediately below for further detail) | Introduction: lines 80 - 95,  Discussion: lines 273-278,  and the Title change |
| b) the ways that spatial analysis itself provides novel insight into patient care, then it is perhaps worthy of reconsideration. | We agree that the notion of using GIS to assess and promote healthcare access has been explored in many areas and have responded to these comments to discuss how this current example adds to the current and previous body of knowledge. We believe this project provides new methodologies that complement and add to the current examples, which focus on using spatial data to assess current healthcare and identifying gaps in care that presently exist. Our clinical planning tool focusses on assessing the differences between a current state (of healthcare service locations) and potential future states that include new locations for healthcare services. Our additions to the discussion section and rewording of the manuscript title aim to address these concerns. | Introduction: lines 80 - 95,  Discussion: lines 273-278,  and the Title change |

**Reviewer 3**

| Comment | Response | Manuscript Changes |
| --- | --- | --- |
| The abstract is well introducing the problem and methodology, but it looks long, try to make it 200-250 words at maximum, also, I think that subtitles are unpopular in the abstract. | We have cut down the abstract and removed the subtitles; the abstract word count is now 245 words. | Lines 21 - 38 |
| This paper is looking more technical paper than a thematic one, which means that the authors don’t discuss a “thematic” paper, like accessibility, distribution…etc, but focus on methods and technics, so it’s preferred to add more technical stuff in material and methods, especially technical staff about software, architecture, source code…etc. | We have added a section in the methods “Analysis Software” which more clearly describes the software (and versions) used in analysis and app development. A link to the source code on GitHub has also been made available, in this section. | Lines 115-124 |
| Because accessibility has multiple definitions and methods to measure either, spatial, functional, acceptability..etc. I suggest adding a literature review about accessibility and related technics used in this paper | We have now included paragraphs in the introduction and discussion to provide more literature review related to defining healthcare accessibility and how this review has factored in the model of accessibility used in this paper. This is intended to add to the discussion about distance measures in the following paragraph (lines 284-297), which is a key aspect of measuring accessibility to services. | Introduction: lines 60 - 79  Discussion: Lines 279-283 |
| it’s recommended to remove white spaces between paragraphs and pages | This has been done |  |

Kind Regards,

Calum Nicholson, MSc.

CHAANZ National Coordinator

Heart Research Institute, Sydney, Australia
