# Supplementary material for: An interactive geographic information system to inform optimal locations for healthcare services
Source: PLOS Digit Health. 2023 May 8;2(5):e0000253. doi: 10.1371/journal.pdig.0000253 (PMC10166531; doi:10.1371/journal.pdig.0000253)
Supplement: Attachment — Submitted filename: REVIEWING An Interactive Geographic Information System to Inform Optimal Locations for Healthcare 4 Services ON PLOS DEGITAL HEALTH REV 1.odt [file pdig.0000253.s002.odt]

REVIEWING An Interactive Geographic Information System to Inform Optimal Locations for Healthcare 4 Services ON PLOS DIGITAL HEALTH REV 1

Dear authors:
I would like to express my gratitude for the method that use to respond to reviewer comments, the table was very good, and I will use your method in my future works.

About the manuscript
The authors take into consideration the previous note about minimising the abstract and removing subtitles, now it looks better, however, in the future try to follow a more straightforward approach which means introducing the problem, after that your approach briefly (no details) and your contributions, finally the outputs and result. Please don’t change your manuscript abstract this time because it's looking good.

The adjusted all notes mentioned before by reviewers (as I see), and he adds more technical details about the software and methods (Opensource are used widely), The data is available online in addition to the application.

I think this manuscript is well enough to accept to pulpish in your journal. I have no furthermore comments. Waiting for other reviewers' decision.

Good luck
